# A Quantitative Analysis of Intraoperative Distractions and When They Occur During General Surgical Operations

**DOI:** 10.7759/cureus.60700

**Published:** 2024-05-20

**Authors:** David Raftery, Shanen Emmanuel, George Ramsay

**Affiliations:** 1 General Surgery, School of Medicine, Medical Sciences and Nutrition, University of Aberdeen, Aberdeen, GBR; 2 Health Services Research Unit, University of Aberdeen, Aberdeen, GBR; 3 Colorectal Surgery, National Health Service (NHS) Grampian, Aberdeen, GBR

**Keywords:** safe surgery, error, non-technical skills, human factors, distraction

## Abstract

Introduction

Distractions in operating theatres prevent team members from concentrating on the complex tasks required for a successful operation. This can be a potential hazard to care for, and previously, correlations have been made between increased theatre distractions and adverse events. However, it remains unclear how frequently such events occur during routine care in theatres. The present study aims to quantify distractions and analyse any differences between staff groups, operative stages, and modes of operation.

Methods

A single-centre prospective study was conducted to assess disruptions in general surgical theatres. Events were recorded using a previously described categorization system on a proforma by a single researcher. The source and severity of distraction were recorded, as well as the mode of operation (elective/emergency), stage of operation, and staff team (scrubbed/floor).

Results

A total of 4,219 minutes of surgery were observed over four weeks, and 1,095 distraction events were recorded. Of the 14 elective and nine emergency procedures recorded, there was a mean of 54.8 distractions per procedure and a frequency of one distraction every three minutes and 51 seconds (15.6 hr^-1^). Irrelevant communication relating to the patient's case was the most common source, accounting for 24.7% of all distractions. The most frequently disrupted stage of the procedure for scrubbed staff was during anastomosis/resection for both elective and emergency procedures, with 16.9 and 32.6 distractions occurring per hour, respectively. Scrubbed staff were significantly more susceptible to distraction in emergency procedures than the floor staff.

Discussion

Our study reflects previous assessments with irrelevant communications and emergency procedures yielding the highest prevalence of distraction. This investigation provides novel information about the different stages of general surgery and the frequency of distractions that occur.

## Introduction

Operating theatres require skilled staff from several disciplines to work in concert to deliver the best outcomes for patients. For this to be delivered effectively, precise, complicated tasks must be completed in a specified sequence, often with minimal room for error. At times, there may be multiple team members present in the theatre, performing the different tasks required of them while also communicating with colleagues [1]. There can be a high level of noise associated with this, and increased background noise can lead to an increased risk of error.

The airline industry demonstrated a link between an increased prevalence of error, "near-misses," and the presence of distraction [2]. Indeed, there has been standardisation across the airline industry to make improvements around human factors contributing to error, including decreased distractions and eliminating irrelevant communication during safety-critical timepoints [3, 4]. Similarly, the World Health Organisation (WHO) has attempted to introduce comparable principles into surgical practice in operating theatres with the use of a 'surgical safety checklist, which includes measures to decrease distractions and identify safety-critical moments, thereby improving patient care and reducing the incidence of adverse events [5].

A distraction is defined as an unnecessary interaction that may cause an interruption to the procedure being performed. Although operative human factors and non-technical skills are relatively poorly studied, distractions correlate to increased adverse events and thus poorer outcomes [6]. Each distraction may lead to an adverse event due to a technical skill error, miscommunication, or inattention. Thus, to decrease the prevalence of errors and near-misses in operating theatres, it is imperative to distinguish where and when distractions occur and if there is a correlation with adverse events. This information could help develop interventions to prevent adverse events and contribute to patient safety.

A recent systematic review of theatre distractions, interruptions, and disruptions showed the negative impacts these factors have on safe surgical outcomes [7]. An analysis of 27 studies correlated high levels of distractions with increased operative times, surgical errors, surgical site infections, and impaired team performance. However, significant knowledge gaps remain regarding morbidity and mortality outcomes due to operative distractions. As McMullan et al. noted [7], current evidence is insufficient to make recommendations about potential useful interventions to address these issues.

Therefore, we aimed to evaluate the quantity and frequency of distractions, including the source and severity, at different surgical timepoints and any associated adverse events to identify where improvements are required. Comparing differences between staff groups and elective and emergency procedures will further strengthen this understanding.

## Materials and methods

Study design

A classification system of distractions previously developed by Healey et al. [8] and altered for this study. A single observer prospective study was carried out on general surgery procedures (Table 1) in Aberdeen Royal Infirmary, Aberdeen, United Kingdom, between November 2, 2021, and November 25, 2021. Informed consent was obtained from the theatre manager and staff present during procedures. Distraction and timepoint events were recorded contemporaneously using Google Forms (Google Inc., Mountainview, CA) and stored on a secure University of Aberdeen Apple iPad (Apple Inc., Cupertino, CA). No patient information was recorded, only events that affected staff were noted. Raw data were transferred to Microsoft Excel (Microsoft Corp., Redmond, WA) for analysis. 

**Table 1:** Theatres and procedures observed n: number of procedures

		Elective (n)	Emergency (n)
Theatres	Theatre W	2	
	Theatre X		2
	Theatre Y	12	
	Theatre Z		4
Open procedures	Oesophagogastrectomy	2	
	Right hemicolectomy	2	1
	Left hemicolectomy	1	
	Subtotal colectomy	1	
Laparoscopic procedures	Cholecystectomy	2	1
	Appendectomy		2
	Right hemicolectomy	4	
Others	Robotic anterior resection	1	
	Transanal minimally invasive surgery (TAMIS)	1	
	Flexi-sigmoidoscopy		1
	Colostomy formation		1

Data collection

A Google Forms proforma was created, classifying the source and severity of each disrupting event on a multi-choice grid. Various stages of general surgical procedures were timestamped, and their safety-critical timepoints were identified as per Table 2. Staff interactions were only deemed and recorded as a distraction if they were (1) unnecessary to task completion, (2) had the potential to or did impact the task, and (3) responded to by a staff member. Interruptions required for the general operative flow and, therefore, necessary were not considered distractions; however, if this interruption was severe and caused delays to the flow of the procedure, it was recorded as 'case relevant'. For example, a surgeon requesting an instrument from the scrub nurses' table was not considered a distraction, but a pause in the operation for instrument procurement outside the sterile area was considered a distraction. 

**Table 2:** Operative timepoints

Timepoints		Start	Finish
Induction		Intravenous anaesthetic infusion	Moving the patient
Positioning		Moving the patient	Final position achieved
Skin preparation		Cleaning of skin	Sterile drapes placed
Abdominal entry and inspection		Knife to skin	Intraabdominal inspection
Mobilisation and division of blood supply		Tissue manipulation	Gut division
Anastomosis/resection		Gut division	Specimen removed/anastomosis
Closing		Final inspection	Abdomen closed
Extubation and emergence		Abdomen closed	Extubated
Operative phase		Knife to skin	Abdomen closed

The severity of the impact of distractions was also recorded, which ranged from 'potentially distracting' to 'flow disrupted'. Theatre staff were divided into two categories. The 'floor' staff were the non-scrubbed staff (nurses, operating department practitioners (ODPs), and support workers), and the 'scrubbed' staff were at the operating table (anaesthetists, scrub practitioners (nurse/department practitioners (DPs)), and surgeons). An example of this would be a phone call, which would initially distract a member of the floor team and then may distract the scrub team if they were interrupted by it or information was communicated with them. Each procedure was recorded, from the induction of anaesthesia to extubating the patient. Therefore, during the induction and emergence of anaesthesia, only the anaesthetist was considered the 'scrubbed' member, and the ODPs and nurses present were considered ‘floor’ staff as they circulated in the theatre.

Ethical review

This study was registered with the Audit with Quality Improvement and Assurance Team of the National Health Service (NHS) Grampian and the University of Aberdeen, Aberdeen, United Kingdom; it was deemed an audit of current practice.

Surgeries

One observer recorded 20 procedures, including elective and emergency cases, across multiple theatres and teams. The same observer was present for all procedures, from induction of anaesthesia to extubation, thus eliminating inter-observer variability. Noteworthy events were recorded in a free text box, and particular attention was paid to the scrub practitioner's instrument, swabs, and sharps counts or any adverse events (such as an unexpected bleed). The length of each section was recorded, along with the various distractions that occurred during this period. The frequency of disruption was calculated by dividing the number of disruptions in a particular operative stage by the time taken for it. This was then further broken down to differentiate between elective/emergency operations and floor/scrubbed staff.

Definitions

As described previously by Healey et al. [8], distractions affecting staff were observed, categorised, and recorded contemporaneously into the described proforma. 

1. Phone: any call or message notification that could be heard in the theatre. Alternatively, if a staff member was using their phone within the theatre; 2. Bleeper: any bleep or pager that could be heard in the theatre; 3. Radio: any music source noted in theatre. Specifically, this refers to instances where staff members requested changes to the music or volume. Included in this was where music was at a high volume and staff communication was hindered; 4. Case-irrelevant communications (CIC): patient-irrelevant, where discussions were irrelevant to the patient in the theatre but regarding other patients/events in the hospital; 5. CIC: medically irrelevant, where discussions referred to issues outside the hospital; 6. Communication difficulties: misheard, repeated, or unanswered communications; 7. External staff: any person entering or leaving the theatre who was not part of the scrubbed or floor team; 8. Equipment provision/failure: any item of equipment that was discovered to be faulty or requesting alternative equipment that caused a delay to operative flow; 9. Working environment: workspace and human-interface issues (e.g., waiting for other staff or equipment to move to complete their own task); 10. Case relevant: disruptions that were necessary to the procedure but caused distractions.

Data analysis

Statistical comparisons were made between the source and time of distractions, categories of staff (scrubbed vs. floor), and modes of operation (elective vs. emergencies). An independent (unpaired), two-tailed Student's t-test was performed on the various parameters using Microsoft Excel. Statistical significance was considered at p <0.05. The frequency of distraction was described as the number of distractions per unit time (per hour, hr^-1^) for clarity and to display data as positive integers.

## Results

**Figure 1:**
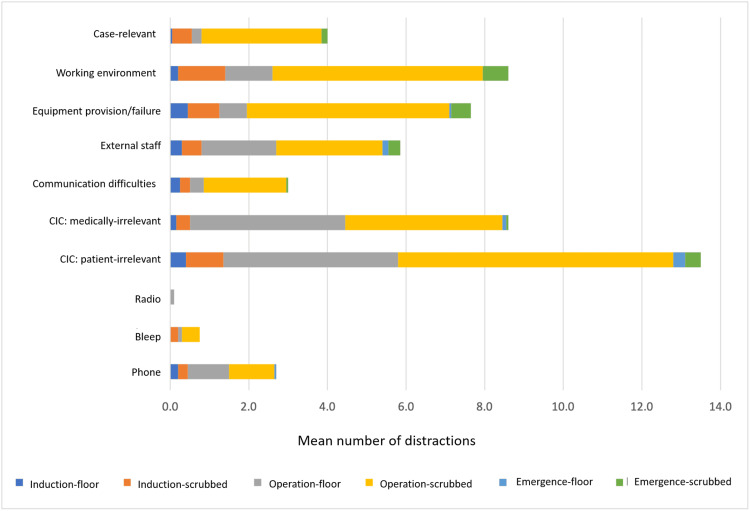
Mean number of disruptions per procedure, when they occur (during induction, operation, or emergence), and whom they affect (floor and scrubbed staff). CIC: case-irrelevant communication

A total of 4,219 minutes of general surgery procedures were observed, and 1,095 distracting events were recorded involving 14 elective and six emergency procedures (Table 1). Case-relevant distractions accounted for 80 (7.3%) distractions across all cases. The mean number of distractions per procedure was 54.8, and the frequency was one distraction per three minutes and 51 seconds (15.6 hr^-1^). The most common distractions affecting the scrubbed team were CIC (patient-irrelevant), working environment issues, and equipment failures/procurement (a per-case mean of 7.0, 5.4, and 5.2, respectively) (Figure 1). The most disruptions in a procedure occurred during the mobilisation and division of blood supply stage, although this was also the longest stage (Table 3). However, the most frequently disrupted stage of an operation, per unit of time, was during the anastomosis and resection stages (Table 4). No serious adverse events were observed; 10 procedures had minor adverse events (minor bleeds, perforations, or organ lacerations that were managed with electrocautery, suturing, or haemostatic agents).

**Table 3:** Mean length and number of distractions for all staff at each timepoint in elective and emergency procedures

Timepoints	Elective		Emergency	
	Length (min)	Distractions	Length (min)	Distractions
Induction	18.8	4.4	17.4	5.2
Positioning	10.4	1.6	13.6	2.2
Skin preparation	6.6	0.9	5.4	1.2
Abdominal entry and inspection	24.2	6.6	13.2	5.4
Mobilisation and division of blood supply	98.9	24.1	40.8	18.8
Anastomosis/resection	40.5	11.4	18.4	10.0
Closing	37.9	9.1	21.8	7.2
Extubation and emergence	18.1	2.1	24.2	5.2
Operative phase	191.1	12.8	81.2	35.0

**Table 4:** Results and analysis CIC: case-irrelevant communication; SD: standard deviation of the mean; N, % denotes the number of procedures and proportion of disruptions; *signifies p<0.05.

		Total	Elective	Emergency
Number of minutes (min)		4219	3429	790
Number of procedures		20	14	6
Mean length of the procedure (min)		211.0	244.9	131.7
Total disruptions (mean ± SD)	All staff	1095(54.8±1.5)	816 (58.3±1.7)	279 (46.5±1.4)
	Floor	334 (16.7±1.1)	224 (16.0±0.9)	110 (18.3±1.7)
	Scrubbed	761 (38.1±1.8)	592 (42.3 ±2.2)	169 (28.2 ±1.0)
Most common disruption source (n, %)	All staff	CIC: patient-irrelevant (270, 24.7%)	CIC: patient-irrelevant (187, 22.9%)	CIC: patient-irrelevant (83, 29.7%)
	Floor	CIC: patient-irrelevant (103, 9.4%)	CIC: patient-irrelevant (51, 6.3%)	CIC: patient-irrelevant (52, 18.6%)
	Scrubbed	CIC: patient-irrelevant (167, 15.3%)	CIC: patient-irrelevant (136, 16.7%)	CIC: patient-irrelevant (31, 11.1%)
Most common disrupted stage (n, %)	All staff	Mobilisation and division of blood supply (432, 39.5%)	Mobilisation and division of blood supply (338, 41.4%)	Mobilisation and division of blood supply (94, 33.7%)
	Floor	Mobilisation and division of blood supply (138, 12.6%)	Mobilisation and division of blood supply (96, 11.8%)	Mobilisation and division of blood supply (42, 15.1%)
	Scrubbed	Mobilisation and division of blood supply (249, 26.8%)	Mobilisation and division of blood supply (242, 29.7%)	Mobilisation and division of blood supply (52, 18.6%)
Frequency of disruptions (hr^-1^)	All Staff	15.6	14.3	21.2
	Floor	4.7	3.9	8.3
	Scrubbed	11.0	10.4	12.9
Most frequently disrupted operative stage (hr^-1^)	All staff	Anastomosis/resection (19.2)	Anastomosis/resection (16.9)	Anastomosis/resection (32.6)
	Floor	Abdominal entry and inspection (6.6)	Induction (5.7)	Abdominal entry and inspection (14.5)
	Scrubbed	Anastomosis/resection (14.4*)	Anastomosis/resection (13.3)	Anastomosis/resection (20.2)

Sources of distraction

**Figure 2:**
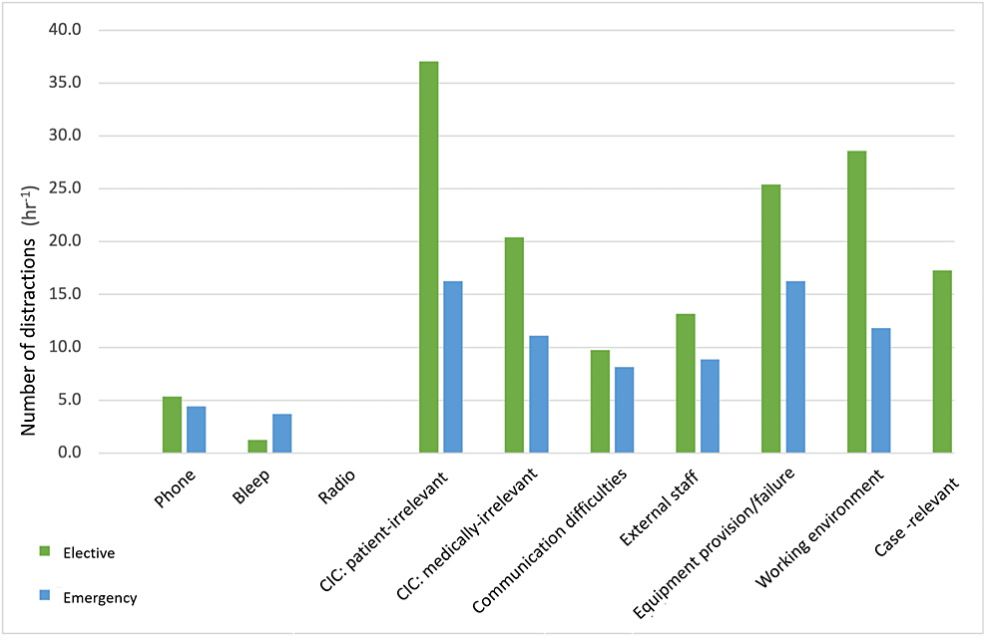
Frequency of distraction sources affecting the scrubbed team during the operative phase, comparing elective and emergency procedures. CIC: case-irrelevant communication

Patient CIC occurred on average 13.5 times throughout a procedure (Figure 1). A total of 270 incidences of this disruption were recorded across the study. Both scrubbed and floor staff were noted to have this type of communication as their most common source of interruption (Table 4). Similarly, this source of disruption was most prevalent for scrubbed staff during the operation phase for elective and emergencies (Figure 2) (a frequency of 37.0 hr^-1^ and 16.3 hr^-1^, respectively). Other than patient CIC, medical CIC was found to be a common distractor. A total of 172 incidences (mean 8.6 per procedure) of medical CIC were recorded; this was the same frequency as working environments sourced distractions. However, there was a more pronounced difference in working environment distractions between elective and emergency procedures for scrubbed staff (p = 0.075) (Figure 2). The least disrupting source was the radio (or other music sources); on only two occasions was the volume considered too loud by a staff member; these were once-per-procedure events and therefore occurred in 10% of procedures.

Times of distraction

**Figure 3:**
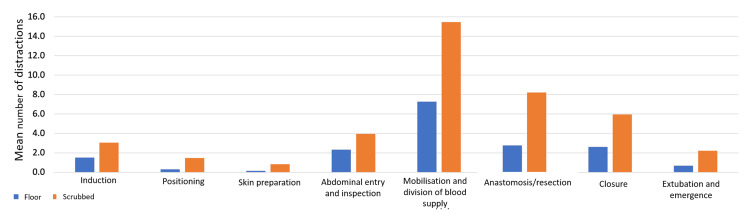
A graph indicating where disruptions occur in procedures and the mean number of distractions for the floor and scrubbed teams

**Figure 4:**
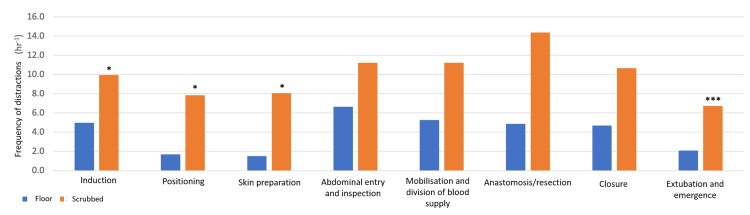
The frequency of distraction (per hour) for the floor and scrubbed teams Statistical comparison to the anastomosis/resection stage for the scrubbed team: * signifies p = 0.05, and *** signifies p <0.001.

For all procedures and staff members, the combined frequency of disruption was 15.6 distractions per hour (0.26 distractions per minute). Overall, emergency procedures had more frequent distractions (21.2 hr^-1^) than electives (14.3 hr^-1^) (p = 0.077). During the emergency procedures, the scrubbed staff had significantly more frequent distractions (12.9 hr^-1^) than the floor staff (8.3 hr^-1^) (p = 0.004) (Table 4). For both teams, the mobilisation and division stages had the most distractions (Figure 3). Notably, the most frequently disrupted section per unit time was the anastomosis/resection stage (Figure 4), which was disrupted 16.9 and 32.6 times every hour in elective and emergency procedures, respectively (Table 4). When this stage was compared to all other stages, it proved to be significantly more distracting (p = 0.0098) (Figure 4). The scrubbed staff were most frequently disrupted during this stage compared to the rest of the procedure (14.4 disruptions per hour). The floor staff were most frequently disrupted during the abdominal entry and inspection portion (6.6 disruptions per hour). When this is separated for the mode of procedure, this was again true for floor staff in the emergency setting (14.5 hr^-1^); however, it was during induction (5.7 hr^-1^) that this was the most frequently disrupted section for floor staff in the elective procedures.

## Discussion

In this novel study assessing the distraction load in a single centre, we demonstrate that the distractions are frequent and varied in terms of their type and severity to the task within the case. Many of the findings in this study correlate with previous assessments of theatre disruptions in analyses of other settings [1]. We see that unnecessary communication (both patient and medical CIC) is the most common source of disruption in the operating theatre.

Here we add to the previous reports by documenting that the mobilisation and division of the blood supply stage are consistently where most distractions occur, but that during the anastomosis/resection stage, the scrubbed staff are most frequently disrupted. This is an important finding, as both of these stages require intense concentration to achieve safe and desired outcomes. A systematic review of 17 published reports of intraoperative distractions [9] showed that CIC and staff movements were the most frequent types of distractions, but equipment and procedural distractions were the most severe, which is likewise reflected in the present study.

A recently published similar study [1] found a comparable frequency of 28.3 distractions per hour with similar frequent sources (CIC, 6.7 hr^-1^), although, in contrast to our work, the staff were not informed about what was being measured. A Glasgow-based, anaesthetic-focused investigation measured the decibel level close to the operating table throughout induction, maintenance, and emergence; similarly, they found frequent CIC in the majority of cases [3]. 

An interesting finding was that the induction stage (5.7 hr^-1^) was the most frequent time when floor staff were distracted during elective procedures. There are many possible suggestions for these findings, which reflect the events occurring in theatre at that time. During the induction stage, the patient is on the table, and the anaesthetist will begin the administration of intravenous anaesthesia, subsequent intubation, and stabilisation of the patient's cardiopulmonary physiology. During this time, there is a minimum number of staff permitted in the theatre, and door signs advise other staff not to enter during this stage. While this is very helpful in creating the ideal environment for the patient and the anaesthetist, it requires the floor staff to manage external staff requests, assist the anaesthetist, and gather the equipment they require. Although the floor team in elective theatres generally has the same individuals, the anaesthetic team tends to rotate and has various preferences in practices and equipment. Furthermore, for some of this stage, the patient is awake, and the floor staff will be their first point of contact and provide them with high-class care.

Suggesting improvements to practice in this situation is challenging as certain factors are beyond the control of staff. Additionally, not all distractions are harmful; some could be viewed as team building, such as staff members chatting during non-safety critical points. Some disruptions are necessary for the procedure to progress; this analysis had 80 such case-relevant incidences, which accounted for 7.3% of the overall disruptions. Teaching moments are difficult to classify as they are not necessarily relevant to the case but necessary to the service in teaching hospitals. In this study, they were only accounted for as case-relevant distractions if they interfered with the procedural flow; however, this was uncommon. 

Similar studies used multiple observers to record distractions [10, 11]. A strength of our study is that we used the same observer to help mitigate inter-observer bias and the potential Hawthorne effect since, after 20 cases, the observer was less likely to be viewed as the 'new' person [12]. However, different individual members of the scrubbed team will have varying levels of permitted background noise during operations, which the floor staff will often be aware of, and this may confound standardisation for analysis. For example, some surgeons were known to request silence in theatres, while others allowed quiet discussion. Limitations to this study include a single centre involving one speciality and the total number of procedures observed. Due to the concurrent COVID-19 pandemic, how we practised healthcare and the quantity and types of cases in surgery changed. This could account for the low number of procedures observed compared to similar studies [11]. However, we believe that our findings are replicable across other surgical specialities and centres.

As mentioned, this study showed that for the scrubbed staff in elective and emergency procedures, the most frequently disrupted portion is during the anastomosis/resection of the gastrointestinal tract. In this period, numerous critical decisions must be made, namely deciding where to dissect or join the bowel and whether it is sufficiently vascularized. These decisions require intense concentration and precise actions rather than frequent distractions. This novel finding was also statistically significant compared to other scrubbed staff stages. Indeed, theatre practises need to introduce steps to limit distractions and their associated adverse events [6]. It may be helpful to use airline procedures such as the 'sterile cockpit' model, whereby distractions are limited during critical stages of the flight, for example, during take-off and landing, to ensure the safe operation of the aircraft. While Kapur et al. [13] and Ricci et al. [14] have discussed that the airline industry and medical fields are not always comparable, this concept has been adapted in critical stages of an operation, for example, during the induction of anaesthesia, and has proven to lead to fewer communication breakdowns in cardiac surgery [15]. The preoperative briefs and safety checklists were consistently performed well across all procedures and provided an opportunity for staff to disseminate and query information. They were perhaps utilising these moments to inform staff of certain sections of the procedure when intense focus is required and to request limited communications and other distractions, for example, the anaesthetist during induction, the scrub practitioner during their counts, and the surgeon during anastomosis/resection. For this to occur effectively, it should be made clear to all staff, students, and visitors when these stages occur, such as when the surgeon asks for a bowel stapler until specimen delivery or when the scrub practitioners are counting aloud. Moreover, this can be extended daily to individual team members sharing what they find distracting or when they require intensive concentration. An operating theatre is a dynamic workplace with highly skilled staff performing intricate tasks to achieve high-quality patient care centred around their safety.

## Conclusions

This work uniquely demonstrates the distraction load within general surgical theatres in a single centre. While it is unclear if the same frequency of distractions occurs elsewhere, this is likely to be the case. Decision-making and concentration are critical to the achievement of complex tasks, and we suggest that reducing the distraction load in this environment would confer patient safety benefits. We highlight areas and timepoints where distractions are high and suggest methods to limit or mitigate them. Furthermore, team training on human error and distraction may be beneficial. Further studies grading the severity of distraction or perhaps the gravity of distraction consequences would be helpful. This may prevent adverse outcomes and improve patient safety. 
